# Generation and Characterization of Six Recombinant Botulinum Neurotoxins as Reference Material to Serve in an International Proficiency Test

**DOI:** 10.3390/toxins7124861

**Published:** 2015-11-26

**Authors:** Jasmin Weisemann, Nadja Krez, Uwe Fiebig, Sylvia Worbs, Martin Skiba, Tanja Endermann, Martin B. Dorner, Tomas Bergström, Amalia Muñoz, Ingrid Zegers, Christian Müller, Stephen P. Jenkinson, Marc-Andre Avondet, Laurence Delbrassinne, Sarah Denayer, Reinhard Zeleny, Heinz Schimmel, Crister Åstot, Brigitte G. Dorner, Andreas Rummel

**Affiliations:** 1 toxogen GmbH, Feodor-Lynen-Str. 35, 30625 Hannover, Germany; weisemann@toxogen.de (J.W.); krez@toxogen.de (N.K.); 2 Biological Toxins, Centre for Biological Threats and Special Pathogens, Robert Koch Institute, Seestr. 10, 13353 Berlin, Germany; fiebigu@rki.de (U.F.); worbss@rki.de (S.W.); skibam@rki.de (M.S.); tanja.endermann@ayoxxa.com (T.E.); dornerm@rki.de (M.B.D.); dornerb@rki.de (B.G.D.); 3 Division of CBRN Defence and Security, Swedish Defence Research Agency (FOI), Cementvägen 20, 90182 Umeå, Sweden; tomas.bergstrom@foi.se (T.B.); crister.astot@foi.se (C.Å.); 4 Joint Research Centre, Institute for Reference Materials and Measurements, European Commission, Retieseweg 111, 2440 Geel, Belgium; amalia.munoz-pineiro@ec.europa.eu (A.M.); ingrid.zegers@ec.europa.eu (I.Z.); reinhard.zeleny@ec.europa.eu (R.Z.); heinz.schimmel@ec.europa.eu (H.S.); 5 Scientific Service of Food-Borne Pathogens, Operational Directorate of Communicable and Infectious Diseases, Scientific Institute of Public Health (WIV-ISP), 1050 Brussels, Belgium; laurence.delbrassinne@wiv-isp.be (L.D.); sarah.denayer@wiv-isp.be (S.D.); 6 Federal Department of Defence, Civil Protection and Sport—Spiez Laboratory, Austrasse 1, 3700 Spiez, Switzerland; christian.mueller@babs.admin.ch (C.M.); stephen.jenkinson@ifik.unibe.ch (S.P.J.); marc-andre.avondet@babs.admin.ch (M.-A.A.)

**Keywords:** botulinum neurotoxin, reference material, recombinant expression, specific activity, characterization, mass spectrometric identification

## Abstract

The detection and identification of botulinum neurotoxins (BoNT) is complex due to the existence of seven serotypes, derived mosaic toxins and more than 40 subtypes. Expert laboratories currently use different technical approaches to detect, identify and quantify BoNT, but due to the lack of (certified) reference materials, analytical results can hardly be compared. In this study, the six BoNT/A1–F1 prototypes were successfully produced by recombinant techniques, facilitating handling, as well as improving purity, yield, reproducibility and biosafety. All six BoNTs were quantitatively nicked into active di-chain toxins linked by a disulfide bridge. The materials were thoroughly characterized with respect to purity, identity, protein concentration, catalytic and biological activities. For BoNT/A_1_, B_1_ and E_1_, serotypes pathogenic to humans, the catalytic activity and the precise protein concentration were determined by Endopep-mass spectrometry and validated amino acid analysis, respectively. In addition, BoNT/A_1_, B_1_, E_1_ and F_1_ were successfully detected by immunological assays, unambiguously identified by mass spectrometric-based methods, and their specific activities were assigned by the mouse LD_50_ bioassay. The potencies of all six BoNT/A1–F1 were quantified by the *ex vivo* mouse phrenic nerve hemidiaphragm assay, allowing a direct comparison. In conclusion, highly pure recombinant BoNT reference materials were produced, thoroughly characterized and employed as spiking material in a worldwide BoNT proficiency test organized by the EQuATox consortium.

## 1. Introduction

Six different *Clostridium* (*C.*) species (Groups I–VI) produce seven different serotypes of botulinum neurotoxins (BoNT/A–G) as their major pathogenic factors [1]. BoNTs constitute the most deadly poisons, as parenteral uptake of nanogram quantities is lethal to human beings. Naturally, an accidental intoxication via food contaminated with BoNT causes the disease botulism, a flaccid paralysis of the striated muscles, finally leading to respiratory failure. Besides, their misuse as a bio weapon, as well as very successful use as licensed drugs for the treatment of many neurological and non-neurological disorders have been described in detail [2,3]. BoNT/A–G occur in many variants called subtypes and mosaic toxins, *i.a.*, due to horizontal gene transfer [1,4]. This large diversity and extraordinary potency complicates their detection enormously [5]. To survive the harsh conditions of the gastrointestinal tract upon oral administration, the 150 kDa BoNTs are accompanied by up to four different, non-toxic neurotoxin-associated proteins (NAPs) in so-called progenitor toxin complexes (PTC) of different sizes (300–760 kDa) [1]. Different variants of a 140 kDa non-toxic non-hemagglutinin protein (NTNHA) form a pH-dependent 1:1 complex (M-PTC) with BoNT, thereby shielding each other against acidic pH and proteolytic degradation [6,7]. Additionally, hemagglutinins form a dodecameric sub-complex, which binds to the M-PTC of BoNT/A1, B, C, D and G yielding the large L-PTC, assists in intestinal adhesion and resorption of BoNT [8,9,10,11]. The BoNTs are classical AB protein toxins, which are produced as single polypeptide chains (sc) and posttranslationally proteolyzed within an 8–22mer peptide loop rich in basic amino acids (AA) to become functionally active. *C. botulinum* of Groups I, III and IV express appropriate proteases, whereas strains of Groups II, V and VI lack this activity and release only scBoNT, which receives its essential activation partially by host proteases. The di-chain BoNT comprises a 50 kDa enzymatically-active light chain (LC) and a 100 kDa heavy chain (HC), which mediates specific receptor recognition on neuronal surfaces, productive uptake and translocation of LC into the neuronal cytosol. Here, the disulfide bridge covalently connecting LC and HC is reduced, the LC liberated and able to specifically hydrolyze one of the three soluble *N*-ethylmaleimide-sensitive factor attachment protein receptor (SNARE) proteins, resulting in blockade of neuroexocytosis [12]. Synaptobrevin/vesicle-associated membrane protein (VAMP)-1 and -2 represent the substrates for BoNT/B, D, F, G and tetanus neurotoxin (TeNT), whereas BoNT/A, C and E cleave the synaptosomal-associated protein of 25 kDa (SNAP-25). In addition, BoNT/C is capable of hydrolyzing the isoforms syntaxin-1A/C, -1B, -2 and -3. Except for BoNT/B and TeNT, hydrolysis occurs in unique positions and requires long stretches of 20–50 residues upstream of the individual scissile bond for optimal cleavage of substrate peptides [13].

While different technologies for BoNT analysis have been established, hardly any universally agreed “gold standards” are available nor have “best practices” been identified in international proficiency tests (PT) [5,14]. Furthermore, no certified reference materials (RM) are currently available, and no inter-laboratory exercises have been performed focusing on the detection of BoNTs from real sample materials. Therefore, it is hardly possible to compare analytical results obtained in different laboratories. Against this background, a central aim of the EC-funded project EQuATox (www.equatox.eu) was to provide an evaluation of existing methods regarding screening and identification of BoNTs by conducting an international PT. Due to the unavailability of qualified BoNT RM, appropriate material had to be produced and thoroughly characterized to serve the BoNT PT [15].

The selection of relevant sero- and subtypes out of the >40 BoNT variants as RMs was guided by available epidemiological data. Statistical analysis of laboratory-confirmed human botulism cases by the U.S., National Botulism Surveillance from 2000 to 2014 revealed that BoNT/A accounted for 50.7%, BoNT/B for 43.5%, BoNT/E for 3.5% and BoNT/F for 1.3% of the 2159 cases (www.cdc.gov). No human cases were attributed to BoNT/C, D and G. Food-borne botulism in the U.S. is predominantly caused by BoNT/A (58.6%) and BoNT/E (28.9%), whereas wound botulism is evoked in 91.7% of cases by BoNT/A and 4.9% by BoNT/B. On the contrary, the major cause of infant botulism is BoNT/B (58.7%), followed by BoNT/A (40.5%) and BoNT/F (0.7%). In summary, BoNT/A, B, E and, to a lesser extent, BoNT/F are relevant for diagnostics of human botulism. In addition, BoNT/C, D and their mosaics produced by *C. botulinum* Group III are dominating in large outbreaks of animal botulism [16,17,18,19]. Therefore, it was decided to produce RM of BoNT/A, B and E with high priority followed by BoNT/F, C and D, whereas BoNT/G was considered to be negligible. With respect to subtypes, in the absence of representative epidemiological data, the corresponding prototype of each serotype was chosen, *i.e*., BoNT/A1, B1, E1, F1, C and D.

Depending on genomic and strain background, as well as environmental conditions, like pH, temperature, redox state, *etc*., the large BoNT family can occur as sc- or di-chain BoNT, as 150 kDa pure BoNT, as 300 kDa M-PTC in varying NTNHA-BoNT combinations or as L-PTCs of up to 760 kDa. This enormous diversity of molecules complicates not only the detection of BoNT in complex matrices, but also the selection of the relevant entity to be produced as RM. The smallest, but fully-functional and well-defined entity constitutes the 150 kDa di-chain BoNT, which was therefore chosen to serve as RM. Sufficient stability of pure BoNT is, e.g., demonstrated by the pharmaceutical product incobotulinumtoxin A comprising only the pure 150 kDa BoNT/A as the active pharmaceutical ingredient. It demonstrates a shelf-life of currently 3–4 years at room temperature in contrast to abobotulinumtoxinA and onabotulinumtoxinA comprising the L-PTC/A with only a 2–3-year shelf-life at 2–8 °C [20]. The BoNT RMs were synthesized by means of recombinant techniques in *Escherichia coli*, which facilitates handling of genetically-modified microorganisms (GMMO) with respect to biosafety and allows reproducible culturing. Furthermore, it improves protein yield due to the overexpression of the gene of interest with optional optimization of codon usage, as well as purity, due to affinity chromatography, and it provides fully-controlled posttranslational proteolytic activation to obtain maximum active BoNT. Subsequently, the highly purified BoNT were characterized thoroughly by various analytical methods with respect to purity, maturation, identity, protein content, *in vitro* catalytic activity, *ex vivo* potency and *in vivo* biological activity.

In this study, highly purified 150 kDa proteins of BoNT/A1–F1 were successfully produced and characterized to serve as RM. They qualified to serve as the spiking material for the conduction of an international BoNT PT organized by the EQuATox consortium [15].

## 2. Materials and Methods

### 2.1. Production of BoNT Proteins

Full-length neurotoxins were produced under biosafety level 2 containment (Project Number GAA A/Z 40654/3/123) recombinantly in K12 *E. coli* strains. Employing the *C*-terminal His6tag, proteins were purified on Co^2+^-Talon matrix (Takara Bio Europe S.A.S., Saint-Germain-en-Laye, France) and eluted with 50 mM Tris-HCl, pH 8.0, 150 mM NaCl, 250 mM imidazole. Employing the *C*-terminal Streptag, proteins were purified on StrepTactin-sepharose matrix (IBA GmbH, Göttingen, Germany) and eluted by 10 mM desthiobiotin (IBA GmbH) in 100 mM Tris-HCl, pH 8.0. For proteolytic activation, BoNT was incubated for 16 h at room temperature with 0.01 U bovine thrombin (Sigma-Aldrich Chemie GmbH, Steinheim, Germany) per µg BoNT. Subsequent gel filtration (Superdex-200 16/60 column, GE Healthcare, Freiburg, Germany) was performed in PBS, pH 7.4. In contrast to all other serotypes, BoNT/F tend to show precipitation and loss of activity upon freeze-thaw cycles, which was resolved by supplementing BoNT/F with 0.1% BSA. Highly purified proteins were shock frozen in liquid nitrogen and kept at −70 °C. Aliquots of the highly purified recombinant BoNT/A, B and E supplied to Robert Koch Institute (RKI) for spiking the BoNT PT samples were supplemented with 0.1% BSA, leading to altered protein concentrations (BoNT/A, 0.093 mg/mL; BoNT/B, 0.017 mg/mL; BoNT/E, 0.124 mg/mL).

For analysis, highly purified proteins were denatured under reducing and non-reducing conditions [21], subjected to 10% SDS-PAGE and detected by Coomassie brilliant blue staining. Additionally, identical samples were run by SDS-PAGE, transferred to a nitro cellulose membrane (GE Healthcare) and detected by mouse anti-His6 monoclonal antibody (mAb) (1:1000; Qiagen, Hilden, Germany), goat anti-mouse-HRP (1:10,000; Thermo Fisher Scientific Germany BV & Co KG, Braunschweig, Germany) and Pierce ECL Western Blotting Substrate (Thermo Fisher Scientific) in a Western blot experiment.

### 2.2. Protein Analysis by Capillary Gel Electrophoresis (CGE)

BoNT purity analysis and quantification were performed with CGE, a semi-automated electrophoresis system (Experion, Bio-Rad, Hercules, CA, USA), according to the manufacturer’s protocol using bovine gamma globulin (BGG) as an external calibration protein. In short, 4 µL of sample were mixed with 2 µL of sample buffer containing detergent and two reference proteins for mass calibration and were loaded to the chip after a short incubation. Native and reduced samples were analyzed in triplicates.

### 2.3. Peptide Sequencing by Liquid Chromatography-Tandem Mass Spectrometry (LC-MS/MS)

Trypsin digests of BoNT were analyzed by LC-MS/MS. Prior to the digestion, the BoNTs were precipitated by methanol as described elsewhere [22], and 50 mM ammonium bicarbonate buffer and a 1:20 weight ratio of sequencing grade trypsin (Promega, Madison, WI, USA) were added directly to the dried protein pellet. No reduction or alkylation was performed, and the samples were digested for 80 min at 45 °C. The reactions were terminated by adding formic acid, and the concentration was adjusted to approximately 200 fmol/µL before LC-MS/MS-analysis. Standard conditions for peptide analysis using a nano-LC coupled to a Q-TOF mass spectrometer (Waters, Milford, MA, USA) were used (described in [22]).

### 2.4. Immunological Detection by Indirect Enzyme-Linked Immuno Sorbent Assay (ELISA)

MaxiSorp microtiter plates (Nunc-Immuno Microwell plate, Sigma-Aldrich, Munich, Germany) were coated with recombinantly-expressed BoNT/A, B, E or F (500 ng/mL) in 50 μL PBS/0.1% BSA overnight at 4 °C and blocked with casein buffer (Diavita, Heidelberg, Germany) for 1 h at room temperature. Following washing, 50 µL of antibody (10 µg/mL; anti-BoNT/A: mouse mAb A709, H_C_A78, A1688 and polyclonal chicken IgY HA29; anti-BoNT/B: mouse mAb B279 and polyclonal chicken IgY HB46; anti-BoNT/E: mouse mAb E136 and polyclonal chicken IgY HE57; anti-BoNT/F: mAb F220 and polyclonal chicken IgY HF58 [23,24,25,26]; polyclonal horse trivalent anti-BoNT/A, B and E Botulism-Antitoxin Behring (Novartis Vaccines and Diagnostics GmbH, Marburg, Germany); “mix”: a polyclonal mouse antiserum detecting BoNT/A, B, E and F (RKI)) were added and incubated for 2 h at room temperature. The ELISA was developed by incubation with HRP-labeled secondary antibody diluted in casein buffer (1 h, room temperature). Finally, the plates were washed five times with PBS/0.05% Tween, and substrate 3,3',5,5'-tetramethylbenzidine (TMB) (SeramunBlau slow, Seramun Diagnostika, Heidesee, Germany) was added. The color reaction was stopped with 0.25 M sulfuric acid, and the absorption was determined at 450 nm (referenced to 620 nm) using a microtiter plate reader (LP400; Anthos Labtec, Wals, Austria).

### 2.5. In-Solution Endopep-MS Assay

The in-solution Endopep-mass spectrometry (Endopep-MS) assay was carried out in a 20 µL reaction volume containing 50 mM HEPES (pH 7.3), 25 mM dithiothreitol, 20 mM (300 µM for BoNT/B) ZnCl_2_, 1 mg/mL BSA, 0.1 mM peptide substrate (for BoNT/A: Biotin-KGSNRTRIDQGNQ-RATR(Nle)LGGK-Biotin (*m/z* 2878.5); for BoNT/B: LSELDDRADALQAGASQ-FETSAAKLKRKYWWKNLK (*m/z* 4037.4); for BoNT/E: IIGNLRHMALDMGNEIDTQNRQIDR-IMEKADSNKT (*m/z* 4041.5); the cleavage site is indicated by a hyphen). Peptides were synthesized by Petra Henklein (Institute for Biochemistry, Charité Universitätsmedizin, Berlin, Germany). BoNT/A or BoNT/E were diluted in HPLC-water to concentrations of 2 ng/µL, 200 pg/µL, 20 pg/µL and 2 pg/µL (BoNT/B: 600 pg/µL, 60 pg/µL, 6 pg/µL and 0.6 pg/µL). One µl of each toxin dilution and one µL of the substrate solution were added to 18 µL reaction buffer, and the different resulting 20 µL solutions were incubated for 17 h (4 h BoNT/B) at 37 °C. Control reactions lacking BoNT were run at the same time as the analytic blank. Cleavage products were further desalted and concentrated with ZipTip C18 resin (Merck Millipore, Darmstadt, Germany), carried out according to the manufacturer's instructions.

MALDI-TOF/TOF-MS: Sample analysis (BoNT/A and E) was done in positive ion reflectron mode utilizing an autoflex speed MALDI-TOF/TOF mass spectrometer (Bruker Daltonics, Bremen, Germany) equipped with a smart beam laser. A one µL sample was mixed with 1 µL MALDI-matrix (12 mg/mL α-cyano-4-hydroxycinnamic acid (Bruker Daltonics) in 0.1% trifluoroacetic acid (TFA) and 70% acetonitrile in water), and 1 µL was deposited on a polished steel MTP 384 target plate (Bruker Daltonics). For matrix suppression, deflection was set to 700; mass spectra were acquired over the mass range *m/z* 700–4200. External calibration was performed with peptide calibration standard II (Bruker Daltonics). Each spectrum is an average of 5000 laser shots. Spectra were processed by flexAnalysis 3.4 software (Bruker Daltonics, 2011). Alternatively, sample analysis (BoNT/B) was done in positive ion reflectron mode utilizing an Axima Confidence MALDI-TOF mass spectrometer (Shimadzu GmbH, Reinach BL, Switzerland). A 2 µL sample was mixed with 18 µL MALDI-matrix (10 mg/mL α-cyano-4-hydroxycinnamic acid in 0.1% TFA and 70% acetonitrile in water), and 0.5 µL was deposited on a gold well plate (Thermo Fisher Scientific, Reinach, Switzerland). Pulse extraction was optimized at 4000 Da; mass spectra were acquired over the mass range *m/z* 1000–5000. External calibration was performed with peptide C104 Peptide Mix (LaserBioLab, Sophia-Antipolis Cedex, France). Each spectrum is an average of 1000 laser shots. Spectra were processed by Launchpad software version 2.9.3 (Shimadzu GmbH, 2011).

### 2.6. Amino Acid Analysis by Liquid Chromatography-Isotope Dilution Tandem Mass Spectrometry (LC-IDMS)

BoNT/A, B and E were hydrolyzed by hydrochloric acid and, thus, inactivated, as verified by the mouse phrenic nerve (MPN) hemidiaphragm assay. AAA was performed as described in Working Instruction D-00604/1 of the Institute of Reference Materials and Measurements (IRMM), based on Muñoz *et al*. [27]. Briefly, the sample is hydrolyzed with 6 M hydrochloric acid in the presence of phenol by using accelerated microwave digestion at 150 °C and high pressure. AA are then separated and detected by LC-MS/MS. Quantification is based on using pure AA for calibration and the isotopically-labeled analogues as internal standards. The purity of the AA calibrants was investigated for the presence of water, organic and inorganic impurities using validated methods. The following deviations from the validated method were made: (1) all pipetting work was performed in a laminar flow cabinet for safety reasons; (2) sample preparation was done by volume using calibrated pipettes and not by weight; calibration solutions, however, were prepared by weight; (3) the volume of toxin, internal standard and buffer solutions *per analysis* was proportionally lowered to be able to perform six repetitions; and (4) an analysis of a sample blank (sample, spiked with isotopically-labeled AA, hydrolysis omitted), usually included as a quality control sample before the analysis of unknowns, was not performed for safety reasons.

### 2.7. Immunological Detection and Quantification by Sandwich-ELISA

For comparison of recombinantly-expressed highly purified BoNT materials with native toxins purified from *C. botulinum* supernatants, commercially available 150 kDa BoNTs (Metabiologics Inc., Madison, WI, USA) were used: BoNT/A1 (Lot #A110911-01; specific activity 2.7 × 10^8^ LD_50_/mg), BoNT/B1 (Lot #B110911-01; 1.2 × 10^8^ LD_50_/mg), BoNT/E3 (Lot #E110911-01; 3.0 × 10^5^ LD_50_/mg; trypsin-activated QC sample 6.0 × 10^7^ LD_50_/mg) and BoNT/F1 (Lot #F110911-01; 1.9 × 10^7^ LD_50_/mg). Prior analysis of the commercial, native BoNT serotypes by RKI had shown a purity of 90%–95%, estimated by SDS-PAGE. The protein concentration was determined by measurement of absorption at 280 nm, 260 nm and 320 nm; however, the individual extinction coefficient ε used for each BoNT serotype was not provided in the data sheet, so the validity of the protein concentration of native BoNT was unclear. Highly purified BoNT/A, B and E generated in this work were tested against the native, commercial BoNT/A, B and E3 in a sandwich-ELISA approach where both materials were used in equivalent concentrations (100 ng/mL–0.01 pg/mL; based on the concentration determined photo-spectrometrically at 280 nm, 260 nm and 320 nm using the Implen^©^ nanophotometer (Munich, Germany) for the commercial material and based on AAA for the recombinant material).

The sandwich-ELISAs were performed using capture and biotinylated detection antibodies followed by a streptavidin-poly-horseradish peroxidase conjugate (PolyHRP40). The following combinations of monoclonal or polyclonal antibodies (coating antibody/detection antibody), BoNT/A, A1688/H_C_A78-biotin; BoNT/B, B279/polyclonal horse trivalent anti-BoNT/A, B and E Botulism-Antitoxin Behring biotinylated; BoNT/E, KE97 (rabbit polyclonal antibody, RKI)/E136-biotin, were used (RKI in-house mAb developed in mice, unless otherwise specified). Briefly, MaxiSorp microtiter plates were coated with primary mAb (10 µg/mL) in 50 µL PBS overnight at 4 °C and blocked with casein buffer (Senova, Jena, Germany) for 1 h at room temperature. Following washing, 50 µL of toxin was added in serial dilutions from 100 ng/mL to 0.01 pg/mL in assay buffer (PBS, 0.1% BSA (Sigma-Aldrich) and incubated for 2 h at room temperature. The sandwich ELISA was developed by incubation with biotin-labeled secondary antibody diluted in casein buffer (1 h, room temperature), followed by washing and detection with Streptavidin-PolyHRP40 (0.5 ng/mL, Senova) and substrate TMB.

### 2.8. Mouse Phrenic Nerve Hemidiaphragm (MPN) Assay

The MPN assay was performed as described previously [28,29]. To limit the consumption of mice, the left and right phrenic nerve hemidiaphragms were excised from female mice of strain RjHan:NMRI (18–25 g, Janvier, St Berthevin Cedex, France) and placed in an organ bath containing 4 mL of Earle's Balanced Salt Solution. The pH was adjusted to 7.4, and oxygen saturation was achieved by gassing with 95% O_2_ and 5% CO_2_. The phrenic nerve was continuously electro-stimulated at a frequency of 1 Hz via two ring electrodes. The pulse duration was 0.1 ms, and the current was 25 mA, to achieve maximal contraction amplitudes. Isometric contractions were recorded with a force transducer (Scaime, Annemasse, France) and the software VitroDat version 3.6.1 (Föhr Medical Instruments GmbH (FMI), Seeheim, Germany). The resting tension of the diaphragm was approximately 10 mN. In each experiment, the preparation was first allowed to equilibrate for 15 min under control conditions. Then, the buffer was changed to 4 mL of Earle's Balanced Salt Solution supplemented with 0.1% BSA, and the toxin-containing solution was added (BoNT/A–F). Toxin concentrations were such to allow the reduction of the contraction amplitude by 50% between 50 and 150 min. The times required to decrease the amplitude by 50% (paralysis time *t*_½_ ≤180 min) at different BoNT concentrations were used to construct the calibration curves for BoNT/A–F. These logarithmic functions were fitted to the calibration curves:

*y* (BoNT/A; 0.408/0.81/1.63 pM) = −16.062Ln(*x*) + 60.648, *R*^2^ = 0.99;
*y* (BoNT/B; 2.40/4.80/9.61 pM) = −10.928Ln(*x*) + 83.967, *R*^2^ = 1.0;
*y* (BoNT/C; 2.73/5.47/10.94/21.88/43.76 pM) = −21.232Ln(*x*) + 137.993, *R*^2^ = 0.994;
*y* (BoNT/D; 0.388/1.16/1.94/3.88 pM) = −18.893Ln(*x*) + 83.032, *R*^2^ = 0.998;
*y* (BoNT/E; 2.04/4.08/8.15 pM) = −23.612Ln(*x*) + 104.11, *R*^2^ = 1.0;
*y* (BoNT/F; 25.77/36.81/73.62/110.4/147.2 pM) = −17.325Ln(*x*) + 143.327, *R*^2^ = 0.998.

Normalized potency was calculated by converting the 70-min paralytic half-time to the corresponding BoNT concentrations employing the above equations and referenced to the least active BoNT/F.

### 2.9. Mouse Bioassay (MBA)

The biological activity of the produced BoNTs was determined in mouse median lethal dose (LD_50_) [30,31]. The mouse LD_50_ bioassay is a quantitative method that determines the dose which would kill half of the tested animals (mice) at day 4 after injecting the toxin into the peritoneal cavity. The unit (U) of biological activity of botulinum toxin is defined as the LD_50_ of the BoNT in a population of mice (1.0 LD_50_ = 1.0 U). Female mice of Charles River Laboratories:Oncins France 1 (Crl:OF1) outbred strain (weighing 18–20 g each) were purchased from Charles River (Chatillon-sur-Chalaronne, France). The test was approved by the Belgian Ethical Committee on Animal Experiment at Scientific Institute of Public Health-Veterinary and Agrochemical Research Centre (IPH-VAR, Reference Number 20130409-01). Received BoNT/A (114 µg/mL), BoNT/B (20.2 µg/mL), BoNT/E (156 µg/mL) and BoNT/F (77 µg/mL) were aliquoted and stored at −80 °C until further use. Each experiment was conducted using a new aliquot of toxin diluted in PBS, 0.1% BSA, pH 7.4. Assays were performed using 5 dilutions of toxin and 10 mice per dose. Dilutions were increased in a geometric progression, and the ratio between successive doses was 1.4. Diluted BoNT samples were administered intraperitoneally to mice (*n* = 10; volume injected per mice was 0.5 mL). Injected mice were observed during 4 consecutive days. Symptoms of botulism and death induced in mice were recorded. The acute toxicity of BoNT was determined by the calculation of LD_50_ [30]. Briefly, the percentage of death among the mice was determined for each dose of toxin. It is assumed that a mouse that survives at a given toxin dose would have survived at a lower dose and that a mouse that dies at a given dose would have died at any higher dose. The total of dead and alive mice and the percentage of mortality are calculated at each dose of toxin injected. The proportional distance from LD_50_ is then calculated from the dilution above and the dilution below the dilution, which gave 50% mortality: proportional distance = (50% − %mortality at dilution below)/(%mortality at dilution above − %mortality at dilution below); log LD_50_ = log of concentration below + (proportional distance × log dilution factor).

## 3. Results

### 3.1. Production of Highly Purified BoNT/A–F

The 150 kDa di-chain BoNT, which can form stable PTCs of varying sizes with the non-toxic NAPs in an environment of pH < 6.5, is the fully-active toxicological entity, is well defined and constitutes the target analyte for unambiguous diagnostic results. Since the composition of high molecular weight PTCs varies depending on sero- and subtype (and corresponding strain), as well as environmental conditions, 150 kDa BoNT/A1–F1 prototypes were chosen to serve as RM (Table 1; for simplicity, subtype nomenclature is not indicated anymore from here on). To ensure the absence of clostridial host proteins, the BoNT RMs were produced by means of recombinant techniques in *E. coli*. This approach also facilitates handling of the GMMO with respect to biosafety and allows reproducible culturing. The coding sequences (CDS) of BoNT/A-F were either amplified by PCR or, if necessary, synthesized with codon usage optimized for expression in *E. coli* [32,33,34]. At the 3'-end, a CDS of His- and/or Streptag affinity tags fused to a thrombin recognition site was added to ensure efficient affinity purification and tag removal. Since only di-chain BoNT displays functional activity in neurons, a posttranslational proteolytic activation of scBoNT is mandatory to obtain maximum active BoNT. For this purpose, the CDS of another thrombin recognition site was inserted into the CDS of the loop region connecting LC and HC [35].

**Figure 1:**
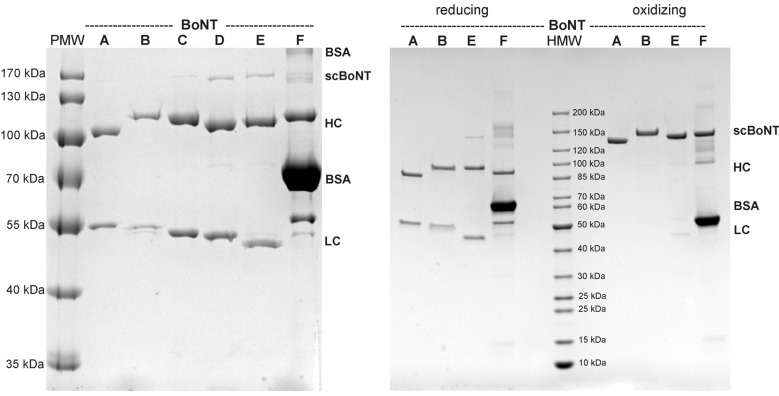
SDS-PAGE analysis of BoNT/A–F. Final products of BoNT/A–F were analyzed under reducing conditions by 10% SDS-PAGE and detected via Coomassie brilliant blue (left panel). All BoNTs displayed ≤12% scBoNT under reducing conditions. One microgram of the final products BoNT/A, BoNT/B, BoNT/E and BoNT/F was analyzed under reducing and oxidizing conditions by 4%–15% gradient SDS-PAGE and detected via Coomassie brilliant blue (right panel). Quantitative formation of the disulfide bridge is demonstrated for all four proteins. The multiple protein bands in BoNT/F are derived from 0.1% BSA as a supplement to stabilize BoNT/F.

All six BoNTs were readily expressed and could be isolated in acceptable to excellent yields as single chain polypeptides by one-step affinity chromatography. Digest with thrombin and subsequent gel filtration yielded highly pure di-chain BoNT/A–F (Figure 1). Western blot analysis using anti-His6tag and anti-Streptag mAbs revealed complete removal of *C*-terminal affinity tags. SDS-PAGE analysis under reducing and non-reducing conditions revealed 88%–100% specific proteolysis into 50 kDa LC and 100 kDa HC (Table 1) and the absence of any degradation products. Incorrect proteolysis outside the loop and, thus not encompassing the disulfide bridge would have resulted in toxin fragments not connected under oxidizing conditions to the remaining BoNT part. Furthermore, the disulfide bridge is quantitatively formed without artificial oxidation, as exemplified for BoNT/A, B, E and F. The theoretical molecular weight of the full-length BoNT ranges from 144.7 kDa (BoNT/E) to 152.9 kDa (BoNT/B) (Table 1). Oxidizing SDS-PAGE resembles this order with the exception of BoNT/A migrating at lower molecular weight (MW) than BoNT/E (Figure 1). Under reducing conditions, the HCs of all BoNTs display a similar pattern around 95–100 kDa. For LC, the main differences in reduced SDS-PAGE compared to theoretical MW are seen for LC/B (too low) and LC/F (too high) (Figure 1; Table 1). The stability of BoNT/A–E in solution was good, but it turned out that BoNT/F tends to precipitate significantly upon freeze-thaw cycles, which was cured by the addition of 0.1% BSA.

**Table 1:** Characteristics of the recombinant BoNT reference material.

BoNT Serotypes	Accession No.		Molecular Weight (MW)			Degree of Activation		Purity	Protein Concentration ± SD			Assigned Protein Concentration	Normalized Potency (MPN Assay)	Biological Activity (LD_50_)	Normalized Biological Activity	Specific Activity
	Gene	Protein	BoNT	LC	HC	SDS-PAGE	CGE	CGE	AAA		SDS-PAGE ^#^					
			(Da)	(Da)	(Da)	(%)	(%)	(%)	(mg/mL)		(mg/mL)	(µM)	-	(pg/mouse)	-	(U/mg)
BoNT/A	M30196	AAA23262	150,266	51,093	99,190	97	99	99.9	0.114	±0.007	n.d.	0.759	123	7.01 ± 0.53	6.9	1.43 × 10^8^
BoNT/B	M81186	AB232927	152,953	52,341	100,630	100	100	96.0	0.0202	±0.0023	n.d.	0.132	19.2	20.22 ± 1.39	2.4	0.49 × 10^8^
BoNT/C	X53751	CAA37780	149,770	51,561	98,226	95	n.d.	n.d.	n.d.	n.d.	0.308	2.054	2.80	n.d.	n.d.	n.d.
BoNT/D	X54254	CAA38175	148,106	51,216	96,907	88	n.d.	n.d.	n.d.	n.d.	0.092	0.620	34.6	n.d.	n.d.	n.d.
BoNT/E	X62089	CAA43999	144,776	48,282	96,512	92	95	93.0	0.156	±0.017	n.d.	1.078	16.2	24.49 ± 0.56	2.0	0.41 × 10^8^
BoNT/F	X81714	CAA57358	148,201	50,053	98,165	90	97	~90	n.d.	n.d.	0.085	0.572	1	48.21 ± 1.13	1	0.21 × 10^8^

^#^ Protein concentrations of BoNT/C, D and F were determined by SDS-PAGE, Coomassie brilliant blue staining and densitometry using BoNT/A, B and E, whose protein concentrations were assigned by AAA as standard proteins. n.d., not determined.

### 3.2. Analysis of Purity, Degree of Activation and Molecular Weight by Capillary Gel Electrophoresis

**Figure 2:**
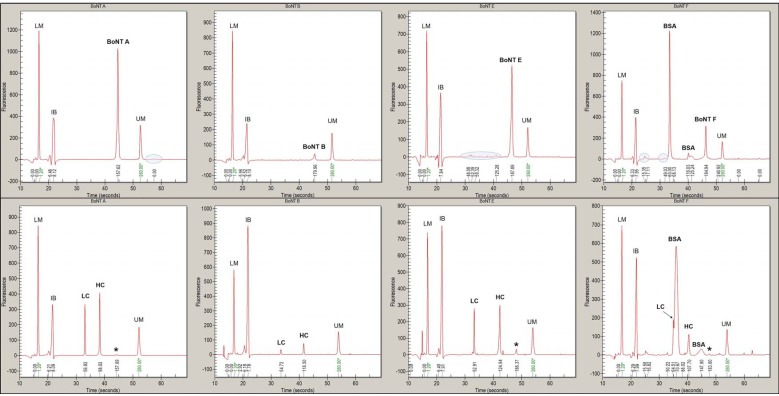
CGE analysis of BoNT/A, B, E and F. Non-reduced (top panel) and reduced (bottom panel) BoNT/A, B, E and F were analyzed by CGE. Contaminations were concluded from repeated analysis and are highlighted in blue circles (top). BSA-related peaks are visible in BoNT/F samples. Trace amounts of un-nicked scBoNT (*; bottom) were detected in BoNT/A, BoNT/E and BoNT/F, whereas BoNT/B was quantitatively hydrolyzed. LM = 1.2 kDa lower marker; IB = instrument background; LC = light chain; HC = heavy chain; UM = 260 kDa upper marker.

The four serotypes BoNT/A, B, E and F pathogenic to humans were given highest priority. Therefore, they were additionally analyzed with respect to purity, degree of activation and molecular weight by CGE. The purity of BoNT/A, B and E ranged from 93% to 99.9% (Table 1). The addition of 0.1% BSA impaired precise analysis of BoNT/F, and its purity was estimated to 90%. CGE analysis of reduced BoNT samples showed that the scBoNT was ≤5% for all four BoNT serotypes (Figure 2 and Table 1), confirming data from SDS-PAGE analysis (Figure 1). In contrast to SDS-PAGE analysis, the deduced MW of oxidized BoNT/B, E and F in CGE was 30–40 kDa higher than the theoretical MW. Upon reduction, all LCs displayed an MW similar to their theoretical reference mass, whereas HC of BoNT/B, E and F displayed an MW increased by 10–25 kDa.

### 3.3. Verification of Protein Sequence Identity by Tryptic Fingerprinting and LC-MS/MS Sequencing

The identity of BoNT/A, B, E and F was verified by tryptic fingerprinting and peptide sequencing by LC-MS/MS. Multiple serotype-specific peptides were identified in all four digestions, and the identified peptides were well distributed over both HC and LC with a sequence coverage ranging from 33% to 50% (Table 2). For BoNT/A, the analysis also revealed parts of the sequence that differ in the recombinant toxin compared to the native one, *i.e*., the loop peptide containing the thrombin cleavage motif at the end of the LC. Furthermore, the BoNT/A1-determining peptide from the LC was detected, containing an alanine at position 26. When using non-reducing conditions during the trypsin digestion, the intact disulfide linkage between the LC and HC of BoNT/A could also be demonstrated, which is in accordance with SDS-PAGE and CGE data.

**Table 2:** Summary of LC-MS/MS results. The identification of BoNT/F peptides suffers from the BSA background in the sample.

Sample	Number of Peptides Identified (of Total)	Identified Peptides in HC	Identified Peptides in LC
BoNT/A	68 (48%)	39	29
BoNT/B	74 (47%)	44	30
BoNT/E	64 (50%)	48	16
BoNT/F	43 (33%)	30	13

**Figure 3:**
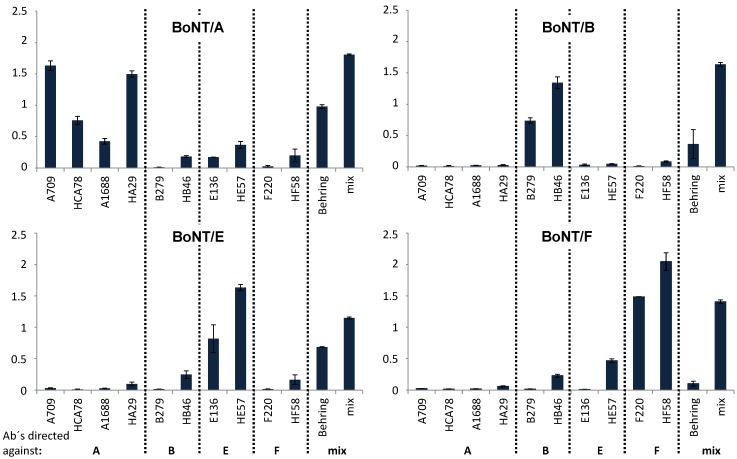
Detection of BoNT/A, B, E and F by indirect ELISA using a set of different antibodies. BoNT/A (top left), BoNT/B (top right), BoNT/E (bottom left) and BoNT/F (bottom right) were coated as the antigen (each 500 ng/mL) in 50 μL PBS/0.1% BSA and tested against different anti-BoNT/A-, B-, E- and F-specific antibodies; antibody names are indicated below the panels, the serotype specificity of the antibodies is indicated by A, B, E or F, respectively. “Behring” is a polyclonal horse trivalent anti-BoNT/A, B and E Botulism-Antitoxin; “mix” indicates a polyclonal serum from mice immunized in parallel with BoNT/A, B, E and F (positive control).

### 3.4. Detection of BoNT/A, B, E and F by Indirect ELISA

A panel of specific polyclonal and monoclonal anti-BoNT/A, B, E and F antibodies was employed to detect the highly purified BoNT/A, B, E and F by indirect ELISA (Figure 3). Overall, the reactivity of the Abs used was as expected from the serotypes tested and from the specificities of Abs, as determined from previous experiments. In particular, the reactivity of the monoclonal antibodies A709, H_C_A78, A1688 (all three directed against BoNT/A), B279 (directed against BoNT/B), E136 (directed against BoNT/E) and F220 (directed against BoNT/F) demonstrated that the sample contained the desired serotype (Figure 3). No evidence for cross-contamination with other serotypes was observed. A few of the polyclonal antibodies showed a certain degree of cross-reactivity between the serotypes, e.g., HF58, a polyclonal chicken IgY directed against BoNT/F, also weakly recognized BoNT/A, B and E, while trivalent anti-BoNT/A, B and E Botulism-Antitoxin Behring also detected BoNT/F, the closest relative of BoNT/E. In the case of HE57, a polyclonal chicken IgY directed against BoNT/E, the most similar serotypes BoNT/A and BoNT/F, but not the much less related BoNT/B, was detected to some extent.

### 3.5. Determination of BoNT/A, B and E Catalytic Activity by Endopep-MS Assay

**Figure 4:**
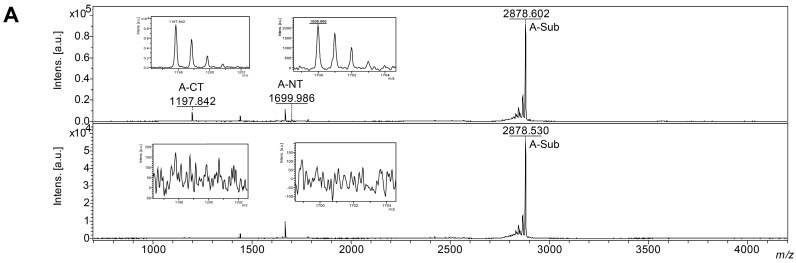

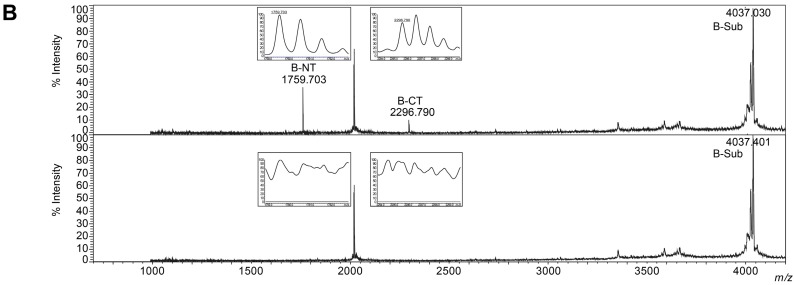

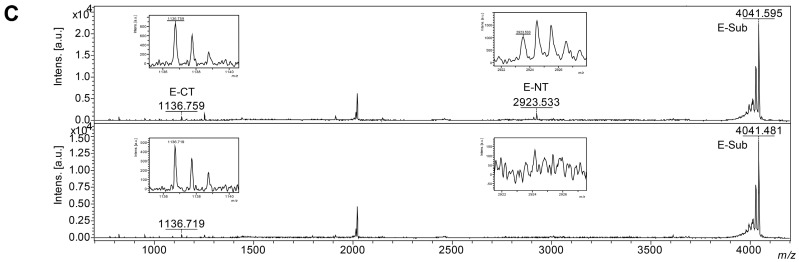
Endopep-MS assay of BoNT/A, B and E. (**A**) Mass spectra of the in-solution activity assay with BoNT/A. Endopep reaction buffer was spiked with 200 pg BoNT/A (upper panel) and without (lower panel). The substrate peak (*m/z* 2878.5), *N*-terminal (*m/z* 1699.9) and *C*-terminal (*m/z* 1197.8) cleaved peptide products from BoNT/A substrate are labeled; (**B**) Endopep reaction buffer was spiked with 60 pg BoNT/B (upper panel) and without (lower panel). The substrate peak (*m/z* 2019.7), *N*-terminal (*m/z* 1759.7) and *C*-terminal (*m/z* 2296.8) cleaved peptide products from the BoNT/B substrate are labeled; (**C**) Endopep reaction buffer was spiked with 200 pg BoNT/E (upper panel) and without (lower panel). The substrate peak (*m/z* 4041.5), *N*-terminal (*m/z* 2923.5) and *C*-terminal (*m/z* 1136.7) cleaved peptide products from the BoNT/E substrate are labeled. Inserts show the isotope peak pattern of cleaved peptides if detectable. The occurrence of only the *C*-terminal peptide fragment in the BoNT/E control sample is due to an instability of this peptide substrate, which was already previously recognized [40,41]. To circumvent this signal, very recently, an optimized peptide substrate for BoNT/E was introduced by incorporating a non-natural homo-arginine residue at P1 position (first AA position upstream of the cleavage site), as well as introducing *C*-terminal amidation of the peptide substrate, which significantly reduced unspecific cleavage and increased peptide stability [41].

The Endopep-MS assay is a rapid and sensitive detection method for differentiating catalytically-active BoNT serotypes [36,37,38,39]. The principle of this method is that BoNTs recognize and cleave a unique site of the synaptic SNARE proteins SNAP-25, Syntaxin -1A/-1B or synaptobrevin/VAMP-1/-2, and the resulting cleaved peptide products are detected by mass spectrometry. Here, Endopep-MS was used to test the *in vitro* catalytic activity of BoNT/A, B and E in serial dilutions on synthetic, truncated peptides. Cleaved peptide products were analyzed by MALDI-TOF/TOF MS (Figure 4). BoNT/A, cleaving the Q^197^-R^198^ peptide bond in SNAP-25, yielded specific cleavage products with *m/z* 1699.9 (*N*-terminal product, Biotin-KGSNRTRIDQGNQ) and *m/z* 1197.8 (*C*-terminal product, RATR(Nle)LGGK-Biotin) (Figure 4A). In contrast, BoNT/B proteolyzes VAMP-1/-2 at Q^76^-F^77^, which results in the cleavage products *m/z* 1759.7 (*N*-terminal product, LSELDDRADALQAGASQ) and *m/z* 2296.7 (*C*-terminal product, FETSAAKLKRKYWWKNLK) (Figure 4B). For BoNT/E, which also hydrolyzes SNAP-25, but between R^180^-I^181^, the specific cleavage products with *m/z* 2923.5 (*N*-terminal product, IIGNLRHMALDMGNEIDTQNRQIDR) and *m/z* 1136.7 (*C*-terminal product, IMEKADSNKT) were identified (Figure 4C). Hence, all three BoNTs exerted their expected specific catalytic activity *in vitro* on artificial synaptic SNARE substrate-based peptides. Similar results were obtained for native BoNT/A, B and E. Endopep-MS analysis of BoNT/A mixed with BoNT/B- and E-specific peptides did not yield any cleavage product. Analogous approaches with BoNT/B and E also showed no sign of cleavage products evidencing the absence of BoNT cross-contamination.

### 3.6. Determination of Protein Concentration of BoNT/A, B and E by AAA

The accurate determination of the amount of protein substance is of crucial importance. Protein concentration determinations based on the measurement of absorption at 278 nm, 260 nm and 320 nm require sufficiently high concentrations in the case of BoNT and the knowledge of the individual extinction coefficient of each BoNT variant. Only for L-PTC/A, an A260/A278 ratio of <0.6 and an extinction coefficient ε (278 nm) = 1.65 mL·cm^−1^·mg^−1^ and ε (280 nm) = 1.54 ± 0.26 mL·cm^−1^·mg^−1^, respectively, are known [42,43], but no value is reported for PTCs of other serotypes or pure 150 kDa BoNTs. In addition to the classical methods by Biuret [44], Bradford [45], Lowry [46] and Kjeldahl [47], protein quantification can be achieved using isotopic dilution mass spectrometry (IDMS) [27]. Here, the BoNT proteins were completely hydrolyzed into their principal components, the AA. Subsequently, six AA (alanine, proline, valine, leucine, isoleucine and phenylalanine) were quantified based on IDMS. Knowledge of the purity and AA sequence composition of the protein of interest allows calculation of the results. The calculations are based on the quantity of individual amino acids present in the solution. The protein preparation to be analyzed with this method needs to be sufficiently pure, otherwise results cannot be related to the theoretical sequence of a given protein and, thus, are meaningless. The protein purity was considered when calculating the concentrations of highly purified BoNT (Table 1): whereas for BoNT/A, the purity assessment by CGE did not reveal any protein (AA containing) impurities in the sample, the CGE purity assessment of the BoNT/B and BoNT/E preparations revealed protein purities of 96% and 93%, respectively (Table 1). BoNT/F had to be excluded due to the addition of 0.1% BSA for stabilization purposes.

### 3.7. Comparison of Recombinant versus Native BoNT/A, B and E by Sandwich-ELISA

**Figure 5:**
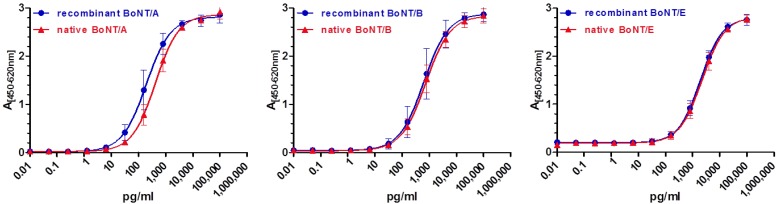
Comparison of recombinant, highly purified BoNT/A, B and E with native BoNT/A, B and E3 using serotype-specific sandwich-ELISA. Coating antibodies and biotinylated detection antibodies were used in a classical sandwich-ELISA format, as described above; the signal obtained by bound antigen was amplified and detected using a streptavidin-PolyHRP40 conjugate and TMB substrate. The absorption was plotted against the logarithmic concentrations of BoNT.

Due to the fact that there is no qualified BoNT RM available, commercial native 150 kDa BoNT/A, B and E3 purified from *C. botulinum* culture supernatant were compared to the recombinant BoNT/A, B and E material produced here by serotype-specific sandwich-ELISA. It has to be kept in mind that the protein concentration of the commercial BoNT determined by the measurement of absorption at 280 nm, 260 nm and 320 nm is not a thoroughly-validated value and was, to the best of our knowledge, not checked by AAA. Still, for an experimental comparison of the recombinant material produced in this work with the native BoNT, the native BoNT, based on the concentration determined by the measurement of absorption, was used in the same concentrations as the recombinant BoNT in serotype-specific sandwich-ELISA. As shown in Figure 5, equivalent concentrations of either native or recombinant BoNT/A, B and E provide very similar results. A precise quantification of the recombinant material *versus* the native BoNT was not performed due to the lack of precise experimental extinction coefficients ε for the differently purified 150 kDa BoNT serotypes and the inaccuracy associated with this value. Instead, AAA was used for precise quantification, since it is known to deliver more precise results than photometric measurement. Therefore, the values obtained by AAA as the most precise concentrations of the highly purified BoNT materials formed the basis for future experiments.

### 3.8. Determination of BoNT/A-F Potency by the MPN Assay

**Figure 6:**
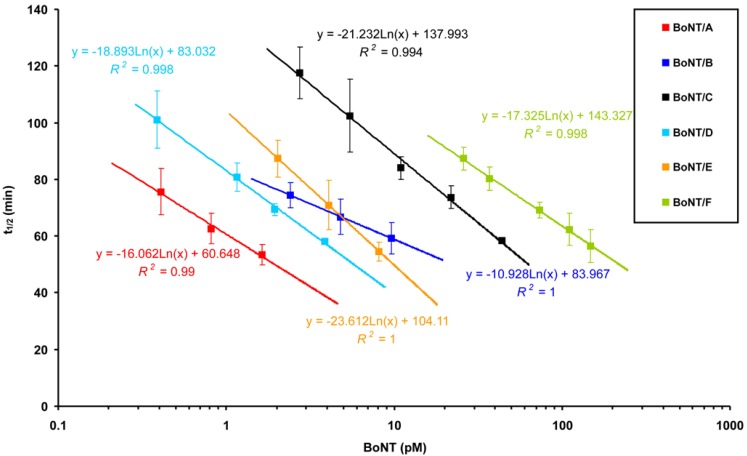
*Ex vivo* mouse phrenic nerve (MPN) hemidiaphragm assay of BoNT/A–F. Concentration response curves were generated based on three to five BoNT concentrations measured in triplicate to quintuplicate. The calculated mean ± SD of the paralytic halftime t_½_ was plotted against the protein concentration. Logarithmic functions were fitted yielding excellent *R^2^* values.

The isolated mouse phrenic nerve (MPN) hemidiaphragm assay is an *ex vivo* method examining the full physiological pharmacodynamic of BoNT by closely reproducing *in vivo* respiratory failure [29,33,48,49,50]. The time period between application of BoNT into the organ bath and halved contraction amplitude correlates with BoNT efficacy and potency, as well as its concentration compared to a BoNT standard material. It has been demonstrated that the paralysis time correlates with the toxicity (MLD, LD_50_, units) determined by the MBA [51]. Here, concentration response curves of all six BoNT/A-F were established, and logarithmic functions were fitted with excellent *R^2^* values (Figure 6). The potency of BoNT/A is the highest, followed by the group of BoNT/D, B and E. BoNT/C and BoNT/F displayed the lowest potency. Employing the logarithmic functions, the concentration of each BoNT serotype causing 70 min paralytic halftime was calculated and normalized to BoNT/F, which displays the lowest potency (Table 1). BoNT/C is ~3-fold more potent than BoNT/F, whereas BoNT/A is 120-fold more potent. BoNT/D is only 3.5-fold less potent than BoNT/A, but 35-fold more potent than BoNT/F. BoNT/B and BoNT/E display medium potencies.

### 3.9. Determination of Biological Activity by Mouse Bioassay

For decades, the MBA determining the median lethal dose (LD_50_) of BoNT in mice has represented the gold standard among various biological, chemical or immunological detection systems for BoNT [5,14]. The MBA has been accepted by pharmaceutical regulatory agencies worldwide to test the potency of BoNT pharmaceuticals [52,53]. However, it comprises some disadvantages: the MBA is costly, lasts for a long period (up to several days in the case of the diagnostics of BoNTs), and most importantly, many mice suffer from botulism and die painfully by respiratory failure due to flaccid paralysis of the diaphragm muscles, which represents its major end point [54]. However, in contrast to the MPN assay, the MBA describes the pharmacodynamics and -kinetics of BoNT. The LD_50_ data presented in Table 1 show the calculated mean LD_50_ ± SD from three independent experiments. These experiments were performed using five dilutions of toxin, a 1.4 ratio between successive doses and 10 mice per dose. The calculation of LD_50_ revealed 7.01 ± 0.53 pg BoNT/A, 20.22 ± 1.39 pg BoNT/B, 24.49 ± 0.56 pg BoNT/E and 48.21 ± 1.13 pg BoNT/F. Again, BoNT/A displayed the highest biological activity; BoNT/B and E exhibited similar and BoNT/F the lowest biological activity. The coefficients of variation (CV) differed between 2.3% and 7.6%, which suggests that the LD_50_ were determined with a high degree of precision.

## 4. Discussion

In light of the dual-use potential of BoNT, the evaluation of existing methods for screening and identification of BoNTs is of major importance to the European Union. Hence, one main aim of the EQuATox consortium was to conduct an international BoNT PT to provide such information, but the task was seriously hampered by the lack of qualified BoNT RM. Therefore, highly purified BoNT had to be generated and qualified as RM to serve as spiking material for the BoNT PT. The recombinant expression of BoNT provides several advantages compared to isolation of BoNT from *C. botulinum* culture supernatants (detailed above). Here, the six serotypes BoNT/A–F were successfully produced in good to excellent yields and purity by similar purification protocols. In addition, the specific proteolysis of the recombinant scBoNT into LC and HC was achieved depending on the serotype almost quantitatively, with a yield of 88%–100%. This is in contrast to partial proteolysis occurring, e.g., in BoNT/B due to its short loop sequence or complete absence of proteolysis in BoNT/E subtypes due to the non-proteolytic *C. botulinum* Group II and *C. butyricum* strains. Quantitative proteolysis allows the direct comparison of all highly purified BoNT with respect to potency and biological activity without significant influences by different culturing conditions, purification protocols and varying degrees of activation.

The four serotypes causing botulism in humans, BoNT/A, B, E and F, were prioritized and thoroughly characterized by various methods. All four highly purified BoNTs were detected and unambiguously identified by means of immunological and different MS-based methods. Validated AAA precisely determined protein concentrations of BoNT/A, B and E, which subsequently allowed the reliable determination of specific activity.

In this study, the normalized potency of BoNT/A determined relative to BoNT/F in the MPN assay is 120-fold higher, whereas BoNT/D is only 3.5-fold less potent than BoNT/A, but 35-fold more potent than BoNT/F. BoNT/B and BoNT/E display medium potencies, while BoNT/C is just ~3-fold more potent than BoNT/F. This order of potency largely resembles that of the specific activity of native purified 150 kDa BoNT/A–F (relative specific activity: BoNT/A 10, BoNT/B 11, BoNT/C 1.8, BoNT/D 10, scBoNT/E 1, BoNT/F 2.4) [55], indicating correct folding and maturation of the recombinant proteins by the developed purification process. Potency is proportional to the affinity and efficacy of a drug; in the case of BoNT, modulation of one of its multiple steps in the mode of action (binding, uptake, translocation and catalytic activity) has been shown to influence its potency in either way [32,35,56,57,58,59]. Along this line, the isolated LC/D and LC/F display similar k_cat_/K_M_ values of ~0.8 µM^-1^ s^-1^ on VAMP-2 [60], but in this study, BoNT/D is 35-fold more potent than BoNT/F, which might be due to differences in binding, uptake and translocation. Even the champion BoNT/A exhibited room for improvement of potency by exchanging its binding domain H_C_ against that of BoNT/B [35].

The native purified 150 kDa di-chain BoNTs all display high specific toxicities, from 10^7^–10^8^ LD_50_/mg of protein with BoNT/E and F at the lower end of the scale, although the relationships might not be the same in different animal species. Comparison of the specific activity of the highly purified BoNT/A, B, E and F with published LD_50_ data (BoNT/A 1.05 × 10^8^ − 1.86 × 10^8^ LD_50_/mg, BoNT/B 0.98 × 10^8^ − 1.14 × 10^8^ LD_50_/mg, BoNT/C 0.88 × 10^8^ LD_50_/mg, BoNT/D 1.60 × 10^8^ LD_50_/mg, BoNT/E 0.21 × 10^8^ − 0.25 × 10^8^ LD_50_/mg, BoNT/F 0.16 × 10^8^ − 0.40 × 10^8^ LD_50_/mg [61]) and data accompanying commercially available BoNT (see Section 2.7) shows that the recombinantly produced BoNTs are at least as active as BoNTs isolated from *C. botulinum*. Quantitative sandwich-ELISA confirmed the equivalence of recombinant and native BoNT/A, B and E.

Native BoNT/F is very often released in minute quantities by *C. botulinum* and displays moderate biological activity. Recombinant expression of BoNT/F overcame such problems for the first time and provided satisfactory amounts of quantitatively-activated BoNT/F, which was confirmed by its high biological activity. However, the stability of the BoNT/F protein was limited upon freeze-thaw cycles and could only be compensated by the addition of 0.1% BSA. Such a proteinaceous stabilizer impeded the characterization by CGE and MS, as well as obviated the determination of the protein concentration by AAA. In the future, the stability and solubility of the BoNT/F molecule have to be improved, and a non-AA based stabilizer compatible with the characterization methods should be developed.

The comparison of potency determined by the *ex vivo* MPN assay with biological activity obtained by the MBA yielded a similar order of functional activity for BoNT/A, B, E and F. In contrast to the 120-fold potency difference between BoNT/A and F determined by the MPN assay, the MBA revealed only a seven-fold difference in specific activity. This difference could be explained by the fact that the *in vivo* MBA describes the pharmacodynamics plus the pharmacokinetics of BoNT, which includes different factors like absorption, distribution, metabolism or elimination from circulation of the individual BoNTs, while factors, such as distribution, metabolism or elimination, are not displayed in the MPN assay.

## 5. Conclusions

In conclusion, highly purified BoNT/A–F were successfully produced by recombinant means and characterized with respect to purity, identity, protein concentration, *in vitro* catalytic activity, potency and specific activity. Results from characterization studies qualified the highly purified BoNT as a reference material suitable to serve as a spiking material for samples to be analyzed in an international BoNT PT organized by the EQuATox consortium [15].

## Abbreviations

AA, amino acid; AAA, amino acid analysis; Ab, antibody; BGG, bovine gamma globulin; BoNT, botulinum neurotoxin; BoNT/X, BoNT serotype X; *Clostridium*, *C.*; CDS, coding sequences; CGE, capillary gel electrophoresis; CV, coefficients of variation; ELISA, enzyme-linked immunosorbent assay; EQuATox, Establishment of Quality Assurance for the Detection of Biological Toxins of Potential Bioterrorism Risk; GMMO, genetically-modified microorganisms; HC, heavy chain; IgY, immunoglobulin Y; kDa, kilo Dalton; LC, light chain; LC-MS/MS, liquid chromatography-tandem mass spectrometry; LD_50_, median lethal dose; L-PTC, large progenitor toxin complex; M-PTC, medium/minimum progenitor toxin complex; mAb, monoclonal antibody; MALDI, matrix-assisted laser desorption/ionization; MBA, mouse bioassay; MPN assay, mouse phrenic nerve hemidiaphragm assay; MS, mass spectrometry; MW, molecular weight; NAP, neurotoxin associated protein; NTNHA, non-toxic non-hemagglutinin protein; PolyHRP40, poly-horseradish peroxidase conjugate; PT, proficiency tests; PTC, progenitor toxin complex; (Q-)TOF, (quadrupole) time-of-flight; RKI, Robert Koch Institute; RM, reference materials; sc, single chain; SDS-PAGE, sodium dodecyl sulfate polyacrylamide gel electrophoresis; SNAP-25, synaptosomal associated protein of 25 kDa; SNARE, soluble *N*-ethylmaleimide-sensitive factor attachment protein receptor protein; TeNT, tetanus neurotoxin; TFA, trifluoroacetic acid; TMB, 3,3′,5,5′-tetramethylbenzidine; VAMP, vesicle-associated membrane protein.
